# New Anti-CGRP Medications in the Treatment of Vestibular Migraine

**DOI:** 10.3389/fneur.2021.799002

**Published:** 2022-01-27

**Authors:** Justin L. Hoskin, Terry D. Fife

**Affiliations:** Department of Neurology, Barrow Neurological Institute, University of Arizona College of Medicine, Phoenix, AZ, United States

**Keywords:** vestibular migraine, vertigo, dizziness, CGRP medication, migraine

## Abstract

**Background:**

Vestibular migraine (VM) is a condition associated with migraine headache, vertigo, dizziness, and balance disturbances. Treatment options are limited. It is unknown if new calcitonin gene-related peptide (CGRP) migraine medications have efficacy in treating VM.

**Methods:**

We retrospectively reviewed all patients with VM who were prescribed one of the new CGRP medications between January 2016 and July 2020. In total, 28 patients met the inclusion criteria. We specifically evaluated the “older” CGRP medications including erenumab, galcanezumab, fremanezumab, and ubrogepant. Medical records for subsequent visits were assessed to monitor improvement described by patients.

**Results:**

Of the 28 patients identified, three were lost to follow up. For the remaining 25 patients, we divided the patients based on a scale of “significant improvement,” “moderate improvement,” “mild improvement,” or “no improvement.” In total 21 of 25 patients demonstrated some level of improvement in their VM symptoms with 15 having moderate to significant improvement.

**Conclusion:**

Results demonstrated a trend toward improvement, suggesting that the CGRP medications appear to be a decent treatment option for VM. A prospective study evaluating CGRP medications in patients with VM would provide further information about this treatment option.

## Highlights

- Vestibular migraine is one of the most common causes of recurrent vertigo.
- CGRP receptors are found in the vestibular system and have potential implications in vestibular disease.
- This retrospective review found that CGRP medications may be beneficial in treating patients with vestibular migraine.
- Future prospective randomized controlled trials may illuminate the potential treatment efficacy of CGRP medications in vestibular migraine.

## Introduction

Vestibular Migraine (VM) is a migraine disorder with associated vestibular symptoms including spontaneous vertigo, positional vertigo, visually-induced vertigo, and head motion-induced vertigo. The term “vestibular migraine” was first introduced in 1999 to describe patients with episodic vertigo related to migraine (1). In 2012, the International Classification of Headache Disorders and Barany Society collaborated to create diagnostic criteria for VM (Table 1).

**Table 1:** Diagnostic criteria for vestibular migraine and probable vestibular migraine adapted from Lempert et al. (2).

**Vestibular migraine**	
A	At least five episodes with vestibular symptoms of moderate or severe intensity, lasting 5 min to 72 h
B	Current or previous history of migraine with or without aura according to the ICHD
C	One or more migraine features with at least 50% of the vestibular episodes: • Headache with at least two of the following characteristics: one-sided location, pulsating quality, moderate or severe pain intensity, aggravation by routine physical activity • Photophobia and phonophobia • Visual aura
D	Not better accounted for by another vestibular or ICHD disorder
**Probable vestibular migraine**	
A	At least five episodes with vestibular symptoms of moderate or severe intensity, lasting 5 min to 72 h
B	Only one of the criteria B and C for vestibular migraine is fulfilled (migraine history or migraine features during the episode)
C	Not better accounted for by another vestibular or ICHD disorder

*Vestibular symptoms include spontaneous vertigo, positional vertigo, visually induced vertigo, head motion-induced vertigo, or head motion-induced dizziness with nausea (2). Vestibular symptoms are moderate or severe if they interfere with daily activities.*

*ICHD, International Classification of Headache Disorders*.

Treatment of VM remains challenging with few randomized controlled trials assessing the efficacy of pharmacologic VM treatments. Several factors contribute to this including lack of standardized nomenclature, incomplete understanding of the pathophysiology, vague and overlapping clinical manifestations, absence of specific tests and biological markers, and only recently developed consensus diagnostic criteria (3). The majority of clinical trials evaluating treatment of VM have been retrospective in nature and results are mixed at best (4, 5). In the last half-decade, several medications have been developed targeting the CGRP molecule or the CGRP receptor for the treatment of acute migraine (6, 7). These medications include erenumab (8), galcanezumab (9), fremanezumab (10), and most recently ubrogepant (11). In our neuro-otology clinic, many patients with VM unfortunately suffer from frequent and severe symptoms despite treatment with more “classic” VM medications. These patients are often desperate for treatment and these new medications appear to be a possible option. Thus, far, no study has been performed to look at the effectiveness of these medications in treating VM.

## Methods

This is a single site retrospective chart review study. We reviewed data on patients diagnosed with VM between January 1, 2016 and July 31, 2020 treated at our facility. Approval for this study was obtained by the institutional review board (IRB number PHX-21-500-035-73-04; 13 August 2020). No funding was required for this study. We hypothesized that a trend toward improvement in VM symptoms would be seen in patients who were started on one of the CGRP medications.

**Figure 1:**
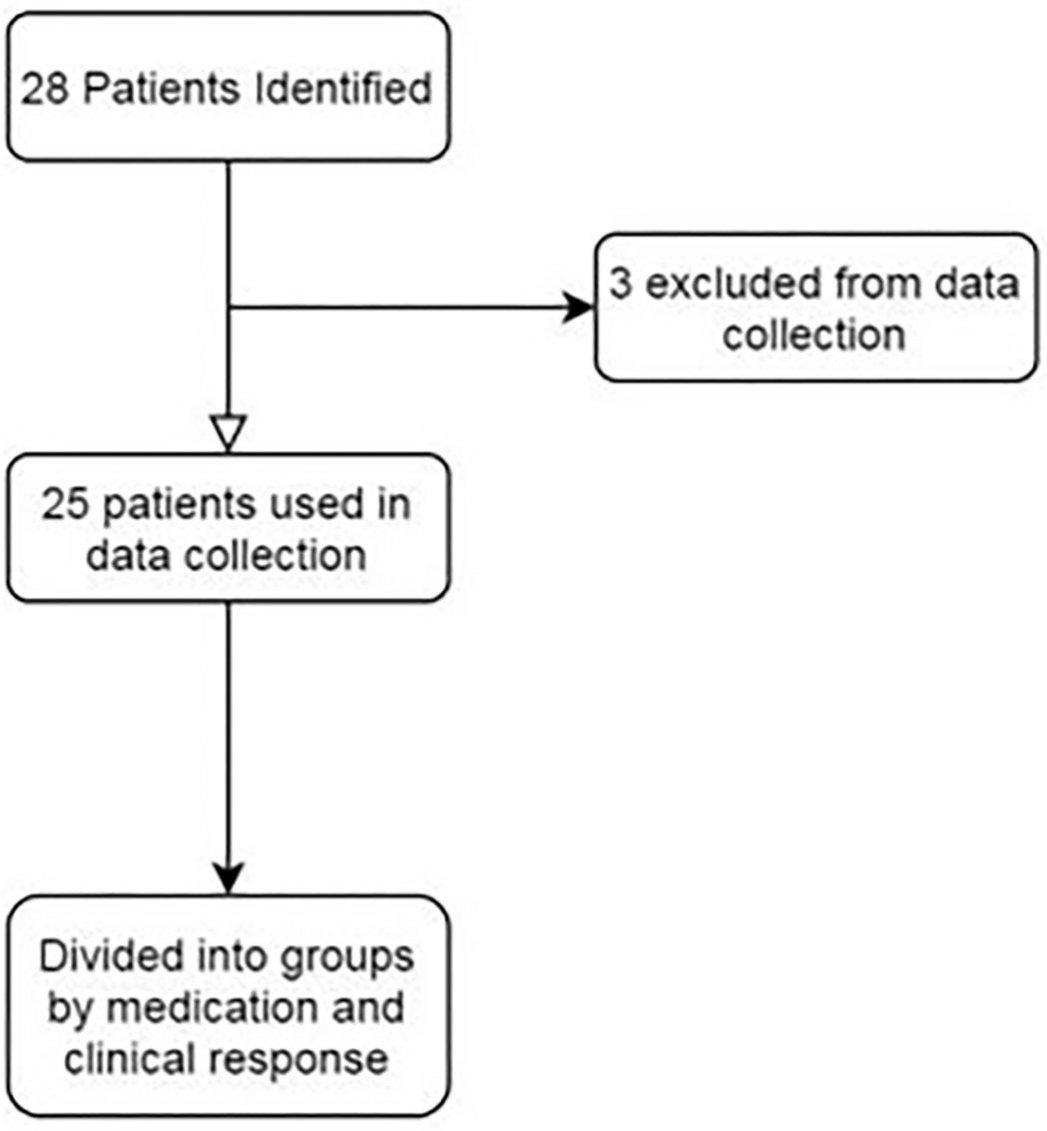
Flow diagram of patient selection, exclusion, and research methodology.

After approval, we searched the medical record for patients who met the following inclusion criteria: diagnosed with VM between the dates previously mentioned, 18 years of age or older, and started on one of the CGRP migraine medications (erenumab, galcanezumab, fremanezumab, or ubrogepant). Patients were likely to be started on the CGRP medications if they had failed other “traditional” migraine medications. The CGRP medications were prescribed to treat primarily the migraine symptoms. We found 28 patients met the inclusion criteria (Figure 1). From there, we collected demographic information about the patients (age, gender, ethnicity, etc) as well as comorbid conditions (migraine, vestibular disorders, anxiety, and depression).

The patients' VM severity before and after starting one of the CGRP medications was recorded. On follow up visits, patients often described improvement as “significant,” “moderate,” “minimal,” or “none.” We divided patients into these categories for ease of data collection and comparison among the different prescribing providers. This was done in part because the providers have different documentation habits making direct comparison challenging. We tracked and recorded whether or not patients described a global improvement in symptoms, meaning their vestibular and migraine symptoms both improved, or if patients described improvement in vestibular or migraine symptoms alone. Patients who did not follow up after the prescription was given were still included in the initial results. Serious adverse reactions were recorded as well.

For the patients included in this study, their information was collected and stored on a secure encrypted network (RedCap). Given the retrospective nature of this project, no patient contact occurred and a waiver of consent was obtained from the IRB.

## Results

We identified 28 patients who met the inclusion criteria listed and recorded basic demographic information (Table 2). Many of the patients (*n* = 21) had concomitant migraine history in addition to the diagnosis of VM. The migraine diagnosis varied from migraine without aura (*n* = 3), migraine with aura (*n* = 1), and/or chronic migraine (*n* = 18). Several patients were found to have a history of a vestibular disorder (*n* = 10) including: peripheral vestibular loss (*n* = 3), benign positional vertigo (*n* = 3), Meniere's disease (*n* = 2), persistent postural perceptual dizziness (*n* = 1), and cerebellar pontine angle meningioma (*n* = 1). Comorbid anxiety (*n* = 11) and/or depression (*n* = 6) was noted with higher frequency. Lastly, we found that on average, patients had tried 5.1 migraine medications prior to initiation of the CGRP medication.

**Table 2:** Demographic characteristics of the participants.

**Characteristics**	**Participants**
	***N* = 28**
Mean age in years (SD)	52.2 (13.5)
Age Groups in years, *n* (%)	
Age <40	6 (21.4)
Age 40–59	12 (42.9)
Age > 59	10 (35.7)
Female, *n* (%)	26 (92.9)
Caucasian, *n* (%)	26 (92.9)
Average duration of symptoms in months	119.4
Comorbid conditions (%)	
Migraine	21 (75)
Vestibular disease	10 (35.7)
Depression	6 (21.4)
Anxiety	11 (39.3)
Number of patients currently or previously on Botox (%)	10 (35.7)

Out of the 28 patients, three were lost to follow up. For the 25 patients who were seen in follow up visits at least once, we reviewed their medical records to identify improvement mentioned by the patient and recorded by the provider. With these results, we divided the patients based on their reported improvement using a simple scale of “significant improvement,” “moderate improvement,” “mild improvement,” or “no improvement” (Table 3). In total 21 of 25 patients demonstrated some level of improvement in their VM symptoms with 15 having moderate to significant improvement. This pattern of improvement was seen in all of the CGRP medications evaluated (Table 3). Patients more often describe a “global improvement” in both vestibular and migraine symptoms. In total, 18 of the 21 patients described this pattern of improvement. The other three patients described improvement in migraine symptoms only, with minimal to no improvement in their vestibular symptoms. Of note, no patient in this small population described improvement in vestibular symptoms alone without change in migraine.

**Table 3:** Results of study.

**Medication**	**Participants (*n*)**	**Seen in follow up (*n*)**	**No longer compliant (*n*)**	**Significant improvement (*n*)**	**Moderate improvement (*n*)**	**Mild improvement (*n*)**	**No improvement (*n*)**
Erenumab	11	9	2	3	3	2	1
Fremanezumab	9	9	2	2	2	3	2
Galcanezumab	6	5	0	3	1	1	0
Ubrogepant	2	2	1	1	0	0	1
Totals	28	25	5	9	6	6	4

*Improvement scale used to best describe improvement seen: significant improvement, moderate improvement, mild improvement, no improvement. Compliance indicates the number of patients who were still taking the medication at follow up. The majority of patients were not compliant with the medication because of no treatment effect. One patient on erenumab stopped the medication because of injection site reaction though they did note mild improvement in vestibular migraine symptoms*.

We also tracked compliance with the medication. All four of the patients who noted “no improvement” had stopped the medication by the time they had a follow up appointment. One additional patient (started on erenumab) stopped the medication despite “mild improvement” because of injection site reaction.

## Discussion

### Background

Vestibular Migraine is recognized as one of the most common causes of spontaneous episodic vertigo (12). The prevalence of VM is estimated to be between 1 and 5% with a recent US population-based survey finding the prevalence around 2.7% (12, 13). In dizziness and headache clinics the prevalence may be closer to 10–30% of patients seen (4, 14, 15).

Consensus guidelines and diagnostic criteria for VM are a relatively recent development with prior descriptions including migrainous vertigo, migraine-associated vertigo, vertiginous migraine, migraine-associated balance disturbance and benign paroxysmal vertigo (5). The prior lack of established diagnostic criteria muddied the waters and made randomized controlled trials difficult to execute, replicate, and critique (5). These diagnostic criteria are closely followed in our clinic, allowing for future replication of this study.

### Pathophysiology

While knowledge on the pathophysiology of VM remains incomplete, most theories resemble those of migraine, with many VM patients having a comorbid long-established history of migraine (4, 5, 14). An in depth discussion of the pathophysiology of VM is beyond the scope of this paper. Suffice it to say that CGRP appears to play an important role. Neuro-anatomically evident connections between the vestibular system and the nociceptive brain stem areas exist, with increased signal transmission between the two systems in patients with VM (16). With the onset of migraine symptoms, there appears to be a change in ion channel function with resultant altered neural activity in the trigeminovascular system (3). This in turn results in release of neurotransmitters like substance P and CGRP (14).

Additionally, receptors for CGRP are expressed in the vestibular system and have been identified as playing a role in motion sickness (17, 18). In migraine patients, there is hyperexcitability of the vestibular system, manifesting in a variety of signs and symptoms including motion sensitivity, motion sickness, and reduced perceptual thresholds of dynamic head movements to name a few (5, 19). Frequently, patients with VM suffer from additional vestibular symptoms; Beh et al. described a study of 131 patients with VM, 61% of which experienced motion sickness (20). They also found that 66% of patients described dizziness with head movements and 51% with constant dizziness (20). It appears clear that CGRP has a role in the vestibular system and in migraine sufferers; targeting this as a possible treatment avenue is worthy of ongoing investigation.

### Treatment

Treatment of VM remains challenging with few randomized controlled trials assessing the efficacy of pharmacologic treatments (21). Due to the paucity of data, the approach to VM treatment tends to follow that of patients with migraine (22). Several prophylactic medications have been assessed including valproate, topiramate, amitriptyline, nortriptyline, propranolol, venlafaxine, flunarizine, and clonazepam to name a few (3–5, 21, 23). The majority of these studies have been retrospective in nature and results are mixed at best (22).

Patients with “chronic vestibular migraine” may not respond to the above medications and can be challenging to treat effectively. Our study primarily evaluated patients prescribed the recently developed migraine preventative CGRP medications: erenumab, galcanezumab, and fremanezumab (10). More recently additional CGRP medications have been released. Thus, far, no study has been performed to look at the effectiveness of these medications in treating VM. These results demonstrated CGRP medications are an option for the treatment of vestibular migraine, providing moderate to significant improvement in 15 of 25 of patients.

Abortive medications certainly play a role in treatment as well and often include medications given for vertiginous symptoms. While we assessed the utility of ubrogepant in this study, the total number of patients evaluated was limited to only two patients. This was in part due to the recent development of this medication in relation to the other CGRP medications assessed. Certainly, ubrogepant has the potential to provide relief, but needs to be studied further. Other medications which can be given for abortive treatment include antihistamines (meclizine), benzodiazepines (diazepam, lorazepam), or antiemetics (promethazine, metoclopramide). Triptan medications have been assessed including zolmitriptan (24), rizatriptan (25), and sumatriptan (26).

### Limitations

This project is retrospective and thus limited in nature and without randomization. We did not compare this patient subset to a control population. One may argue that some of the improvement seen was a function of the passage of time. However, the vast majority of these patients suffer from chronic disease, and so this may not be much of a confounding variable.

By the nature of the diagnostic criteria for VM, patients often have a diagnosis of migraine as well (migraine with or without aura, chronic migraine). Future studies should tease out the severity of migraine symptoms in an attempt to control this aspect of the patient history. There may be value in attempting to treat “vestibular predominant” VM, but these patients may end up with another diagnosis like benign recurrent vertigo, etc. The treatment effect of CGRP medications on these “non-migrainous” conditions may need to be assessed separately.

Each CGRP medication was not evaluated individually, rather the group was seen as a whole. Future prospective studies would obviously need to single out one medication for a more accurate assessment of efficacy. Drop out was present with three out of 28 (10.7%) lost to follow up.

## Conclusion

CGRP medications appear to have a role in the treatment of VM. Further prospective studies are required to fully assess the efficacy of these medications for VM. We anticipate that they may serve as an invaluable option in patients with chronic and severe VM. With the recent updated clinical criteria, prospective studies will be more replicable and better suited for application of results to patients at large.

## Data Availability Statement

The raw data supporting the conclusions of this article will be made available by the authors, without undue reservation.

## Ethics Statement

The studies involving human participants were reviewed and approved by IRB PHX-21-500-035-73-04. Written informed consent for participation was not required for this study in accordance with the national legislation and the institutional requirements.

## Author Contributions

JH: analysis and interpretation of data and drafting the manuscript. TF: study concept and design of the study. Both authors assisted with drafting the manuscript for intellectual content and read and approved the final manuscript.

## Conflict of Interest

The authors declare that the research was conducted in the absence of any commercial or financial relationships that could be construed as a potential conflict of interest.

## Publisher's Note

All claims expressed in this article are solely those of the authors and do not necessarily represent those of their affiliated organizations, or those of the publisher, the editors and the reviewers. Any product that may be evaluated in this article, or claim that may be made by its manufacturer, is not guaranteed or endorsed by the publisher.
